# CCN1 as a compartment-specific biomarker of active microenvironmental remodeling: evidence from bone repair, aging, inflammation, and cancer

**DOI:** 10.3389/fmed.2026.1843283

**Published:** 2026-08-18

**Authors:** Angela B. Tran, Greisha L. Ortiz-Hernandez

**Affiliations:** 1 Department of Medical Sciences, College of Health Sciences, Western University of Health Sciences, Pomona, CA, United States; 2 Division of Biomarkers of Early Detection and Prevention, Department of Population Sciences, City of Hope Comprehensive Cancer Center, Duarte, CA, United States

**Keywords:** angiogenesis, bone pathology, cancer, CCN1, tumor microenvironment

## Abstract

Cellular communication network factor 1 (CCN1) is a secreted matricellular protein induced by growth factors, mechanical stress, hypoxia, and tissue injury and regulates diverse cellular processes through interactions with integrins, heparan sulfate proteoglycans, and extracellular matrix (ECM) components. Although prior reviews have broadly summarized CCN1 biology, its potential as a biomarker warrants a more focused synthesis of empirical evidence. Here, we propose that CCN1 should be viewed not as a generic disease marker, but as a compartment-specific biomarker of active microenvironmental remodeling. Emerging evidence identifies CCN1 as a central regulator of bone pathology and cancer progression, functioning at the intersection of osteogenesis, osteolysis, angiogenesis, stromal remodeling, immune modulation, and metastatic colonization. Within the skeletal niche, CCN1 is produced by tumor cells, stromal fibroblasts, endothelial cells, and bone-resident cells, where it influences osteoblast and osteoclast activity and promotes vascular niche formation. CCN1 also participates in fracture repair, bone marrow mesenchymal stem cell aging, epithelial–mesenchymal transition, and tumor dissemination, highlighting its roles in both regenerative and pathological processes. Importantly, CCN1 exists in distinct biological compartments, including ECM-bound, soluble circulating, extracellular vesicle-associated, and tumor cell-associated pools. These compartments likely convey different biological information, ranging from local osteogenic to systemic injury, inflammation, and metastatic spread. While circulating CCN1 is measurable and shows promise as a minimally invasive biomarker, its interpretation must account for disease context, timing, and biological compartment. We conclude that CCN1 represents a compelling translational candidate linking bone remodeling and cancer biology, with future studies needed to validate its biomarker utility in skeletal disease and metastasis.

## 1 Introduction

The bone is a dynamic, highly vascularized tissue that consistently undergoes remodeling via osteoclast resorption and osteoblast formation (1, 2). This remodeling process creates a permissive microenvironment for tumor colonization because cancer cells can exploit programs normally used for repair, vascularization, immune cell trafficking, and matrix turnover. The cellular communication network factor 1 (CCN1), one of the first proteins discovered in the CCN family, is positioned at this interface (3–5). CCN1 is an immediate-early gene that is activated without additional signals from *de novo* protein synthesis and coded for secretory proteins including growth factors and putative proteins (6). It is also widely known as a secretory matricellular protein with diverse implications, such as cell extracellular matrix (ECM) communication, angiogenesis, and chondrogenesis (5–7).

CCN1 is a secreted matricellular protein with four conserved domains: insulin-like growth factor binding protein (IGFBP)-like domain, a von Willebrand factor type C (VWC) domain, a thrombospondin type I repeat (TSP1), and a C-terminal cystine knot motif (3, 8, 9). These domains allow CCN1 to function as a molecular scaffold with integrated signals in the ECM since it can bind integrins, heparan sulfate proteoglycans, and growth factors (10, 11). Although fundamentally CCN1 is a secretory protein it exists in two distinct forms: ECM-bound or soluble. This creates distinct pools of CCN1 that are either bound or soluble, and this is biologically significant since CCN1 can locally regulate adhesion in its ECM-bound form or have a hormone-like function in its soluble form (5, 12). CCN1 main cellular functions are achieved through binding to heparan sulfate proteoglycans and integrins, including αvβ3, α6β1, and αMβ2. These interactions activate downstream signaling pathways such as ERK, FAK, PI3K, and NF-κB, regulating cell migration, survival, and differentiation (7, 10, 13).

In this mini review, we synthesized emerging empirical evidence to evaluate CCN1 as a biomarker of active remodeling, rather than as a static marker of disease presence. By supporting evidence showing that CCN1 is temporally regulated during fracture repair, depleted from aged bone marrow ECM, secreted by tumor cells, detected in blood and linked to inflammatory and vascular injury states. In addition, we caution against overinterpretation, understanding that CCN1 role is context-dependent, and its biological function differs depending on whether it is measured in tissue ECM, plasma/serum, tumor cells, or circulating tumor cells.

## 2 CCN1 compartments define biomarker interpretation

A major barrier to translating CCN1 into clinical use is that it exists in both ECM-bound and soluble forms, and these pools have distinct biological roles. Matrix-tethered CCN1 regulates local tissue remodeling, adhesion and mechano-transduction and repair signaling. Soluble CCN1 in plasma or serum may reflect secretion, proteolytic release, vascular injury, inflammation, or tumor-associated release (14, 15). Additional evidence supports that CCN1 detected in circulating tumors cells may instead identify a cell state associated with mesenchymal features, survival under stress, and dissemination as its expression is minimal in normal blood cells but markedly elevated in tumor cells with mesenchymal characteristics (12). These distinctions are critical for biomarker development because a tissue-associated injury signal may not behave like a stable systemic analyte across every disease state.

CCN1 compartmentalization should be interpreted as a dynamic process rather than a fixed binary state. The proportion of CCN1 that remains ECM-associated versus soluble is likely influenced by secretion rate, ECM composition, availability of integrins and heparan sulfate proteoglycans, proteolytic remodeling, and local mechanical or inflammatory cues. CCN proteins are modular matricellular scaffolds that interact with ECM proteins, growth factors, and cell-surface receptors, allowing them to organize local signaling networks rather than functioning only as freely diffusible cytokines (9, 14, 15). In matrix-rich or mechanically active microenvironments, such as remodeling bone, fracture callus, fibrotic stroma, or metastatic niches, newly secreted CCN1 may be preferentially retained in the ECM, where it supports integrin-dependent adhesion, mechanotransduction, angiogenesis, and repair signaling. Conversely, soluble CCN1 may increase when secretion exceeds matrix-binding capacity, when proteolysis or ECM remodeling releases matrix-associated CCN1, or when vascular injury, inflammation, tumor secretion, or extracellular vesicle-associated transport increases systemic availability.

This model is further complicated by the broader behavior of CCN family proteins. CCN members share conserved modular structures, can mutually reinforce or neutralize one another’s functions, and may act in context-dependent combinations within the tumor microenvironment (14–16). The carboxy-terminal cystine-knot-containing domain has been described as a potential dimerization module, raising the possibility that multimeric or combinatorial CCN interactions may alter ECM retention, receptor engagement, and signaling output (16). Therefore, future biomarker studies should distinguish total soluble CCN1 from ECM-associated, extracellular vesicle-associated, cell-associated, and potentially higher-order CCN-containing complexes.

This compartmental model also reconciles apparently conflicting observations. CCN1 is measurable, remarkably stable in plasma and resistance to freeze–thaw degradation, making it well suited for clinical workflows (17). In specific, a liquid biopsy study focused on early detection of breast cancer demonstrated that CCN1 is stable in plasma with almost the same concentration after freeze–thaw cycles as sample collection, showing strong pre-analytical stability (17). In contrast, foundational studies describe CCN1 as an immediate-early gene, and UV-irradiated fibroblasts show transcriptional induction of CCN1 through the activator protein-1 (AP-1), consistent with dynamic regulation after tissue stress (18). Thus, CCN1 may be measurable and stable in stored plasma while remaining biologically dynamic *in vivo*. Biomarker studies should therefore distinguish analytical stability from biological half-life or turnover.

## 3 CCN1 in skeletal repair and bone aging

Fracture repair provides one of the clearest empirical models for CCN1 as a temporally regulated remodeling signal. Hadjiargyrou and colleagues identified CCN1 among genes upregulated in post-fracture callus and showed that expression increased during the reparative phase, particularly in fibrous tissue, periosteum, proliferating chondrocytes, osteoblasts, and immature osteocytes (19). This pattern supports a role for CCN1 in cartilage and bone formation during repair rather than a nonspecific association with bone tissue.

In an ovine tibial osteotomy model, Lienau and colleagues found that CCN1 protein expression was upregulated early during fracture healing and decreased over time. Reduced fixation stability was associated with lower CCN1 immunoreactivity, reduced vascularization, and delayed healing (20). In osteoblast-centered experiments, Athanasopoulos and colleagues showed that VEGF induced CCN1 mRNA and protein in osteoblasts, that CCN1 contributed to endothelial migration and sprout formation, and that blockade of CCN1 impaired fracture healing in mice (21). In recent studies, Lang and colleagues mapped endogenous CCN1 during bone repair and tested recombinant CCN1 delivery in a mouse femoral fracture model. Here, CCN1 delivery enhanced angiogenesis and accelerated neovascularization, although effects were disrupted by early mechanical loading (22). Collectively, these studies support CCN1 as a marker and mediator of the angiogenic phase of skeletal repair.

Bone marrow mesenchymal stem cell aging provides a complementary example of ECM-associated CCN1 as a niche biomarker. CCN1 is abundant in the ECM produced by young bone marrow mesenchymal stem cells (BM-MSCs) but markedly reduced in aged BM-MSCs, paralleling age-associated declines in osteogenic capacity (23). CCN1 bone-specific knockout mice were utilized to investigate the effects of CCN1 on osteoblast and osteoclast function. Depletion of CCN1 from the bone ECM impaired osteoblast and osteocyte activity and resulted in reduced bone mass and defective skeletal homeostasis in mouse models, underscoring its requirement for normal osteogenesis (24). In addition, Marinkovic and colleagues compared ECMs produced by young and elderly bone marrow stromal cells and found that CCN1 was present in young ECM but undetectable in elderly ECM. Genetic depletion of CCN1 from young ECM reduced the ability of the matrix to support BM-MSC proliferation and growth factor responsiveness, whereas increasing CCN1 incorporation into elderly ECM restored responsiveness to osteogenic factors such as BMP-2 and IGF-1 (23). These studies directly support the statement that CCN1 is enriched in young BM-MSC-derived ECM, reduced with aging, and it places CCN1 within a functional bone marrow niche.

## 4 CCN1 as a cancer-associated biomarker

The strongest biomarker evidence for circulating CCN1 in cancer currently comes from breast cancer studies. CCN1 is detectable in circulating tumor cells yet absent from normal blood and bone cells in healthy donors (12). Bartkowiak and colleagues analyzed soluble CCN1 in plasma from patients with breast cancer and healthy controls using ELISA and reported high specificity and sensitivity for cancer detection in their cohorts, including detection of small T1 cancers (17). The same group also reported CCN1 expression in disseminated tumor cell lines and in a subset of circulating tumor cells, while healthy blood and bone marrow cells were CCN1-negative in their assays (12). Functionally, CCN1 knockdown reduced tumor cell viability and was associated with altered HIF-1α expression and apoptosis (12, 25, 26), suggesting that CCN1 is not merely a passive marker but may mark cells with survival capacity during dissemination.

Loss of CCN1 reduces tumor cell viability, indicating a functional survival role (27, 28). CCN1 expression increases with metastatic progression (29, 30), and its stability in plasma supports development of liquid biopsy strategies (8, 17). Aggressive liquid cancer subtypes show elevated CCN1 expression (12, 31), and CCN1 is detected in disseminated tumor cell lines, circulating tumor cells, and overt bone metastases. The convergence of cell line and clinical data strongly supports a functional role for CCN1 in metastatic competency.

CCN1 acts as a context-dependent marker of invasive tumor promoting microenvironments. Specifically, CCN1 supports angiogenesis by supporting endothelial migration and survival through integrin-dependent signaling, particularly via integrins αvβ3 and α6β1 pathways to activate ERK/FAK/PI3K signaling that supports neovascularization and protect cells from apoptosis (5, 32–34). Because α_*V*_β_3_ integrin is a key regulator of osteoclast activity and angiogenesis within bone, this signaling axis links CCN1 to both osteolytic remodeling and metastatic vascular support. In breast cancer models, CCN1 and αvβ3 integrin signaling regulate survival, proliferation, and chemoresistance through ERK1/2 signaling, and CCN1 has been linked to invasive breast cancer phenotypes and S100A4-associated invasion. Instead, suppressing CCN1 diminishes ERK activation, S100A4 expression, and invasiveness (32, 35–39).

CCN1-mediated angiogenesis is dependent on favorable mechanical conditions, and its delivery enhances angiogenesis to improve regenerated bone strength during osteogenesis (22, 40). CCN1, a direct YAP/TAZ target and is highly expressed in stromal vascular composites during mechanically regulated bone repair (22, 41). Because tumor growth requires similar vascular support, tumor-secreted CCN1 can precondition skeletal niches by enhancing aberrant neovascularization. For example, in pancreatic ductal adenocarcinoma, tumor-secreted CCN1 acts downstream of Sonic hedgehog (SHh) signaling to drive aberrant neovascularization (42, 43). Since bone remodeling is tightly coupled to vascular invasion, this microenvironmental specificity supports a model in which tumor-derived CCN1 preconditions skeletal niches for metastatic colonization. In pancreatic cancer also, CCN1 promotes epithelial–mesenchymal transition (EMT), invasion, and tumor stemness (44, 45). CCN1 expression varies across pancreatic cancer cell lines and correlates with aggressiveness (44), linking CCN1 signaling to metastatic potential. These studies support a mechanistic biomarker framework in which CCN1 reports remodeling states, such as angiogenic, stromal, immune, and survival associated.

## 5 Inflammatory and vascular disease evidence supporting CCN1 biomarker specificity

Findings from non-cancer disease contexts provide important insight into the specificity of CCN1 and help delineate its utility as a biomarker of cancer and bone-related processes. Patients with rheumatoid arthritis (RA) exhibit significantly elevated serum CCN1 compared with healthy controls, however, disease activity showed an inverse relationship with serum CCN1 in the reported cohort (15, 46). Similar inverse relationships have been reported in colorectal cancer progression (46, 47). These findings support clinical measurability but warn that directionality may not be intuitive and may change with disease activity or treatment response.

In lupus-associated pulmonary hypertension, higher CCN1 levels correlate with improved survival, suggesting potential protective or compensatory roles depending on context (48). Circulating CCN1 correlates with inflammatory cytokines and pulmonary inflammation in chronic obstructive pulmonary disease, since circulating CCN1 correlates with inflammatory cytokines and declining lung function. This supports its role as an active systemic mediator rather than a passive byproduct (49, 50). In cardiovascular disease, CCN1 is highly expressed in atherosclerotic lesions and ischemic cardiomyopathy, particularly in vascular smooth muscle–rich regions and has also been studied in its circulating and thrombus-associated forms as a potential biomarker of coronary artery disease and acute myocardial injury (51, 52). These observations highlight the complex and context-dependent biology of CCN1, supporting its value as a dynamic biomarker of active tissue injury and remodeling while suggesting that its clinical specificity may require integration with complementary biomarkers and disease-specific clinical stratification.

For bone pathology and skeletal metastasis, the limited disease specificity of CCN1 should not necessarily be viewed as a weakness, but rather as a reflection of its biological role in coordinating tissue repair and microenvironmental remodeling (53). CCN1 is dynamically induced during inflammation, wound healing, angiogenesis, fracture repair, and extracellular matrix remodeling, processes that are shared across diverse physiological and pathological conditions. Accordingly, elevated circulating or tissue-associated CCN1 may be most informative when interpreted as a marker of active remodeling states rather than as a disease-specific indicator alone. In the skeletal setting, altered CCN1 expression may reflect ongoing vascular, stromal, inflammatory, or matrix remodeling associated with fracture healing, chronic inflammatory disorders, metastatic niche formation, or therapeutic response (54). Furthermore, the ability of CCN family proteins to regulate osteogenesis, angiogenesis, and tumor–bone interactions support their relevance as indicators of dynamic changes within the bone microenvironment (55). Consequently, future translational studies should evaluate CCN1 as part of multimodal biomarker panels rather than as a stand-alone diagnostic marker, integrating soluble CCN1 measurements with complementary imaging modalities, bone turnover markers, inflammatory biomarkers, and tumor-derived analytes to improve biological interpretation and clinical specificity (56) (Table 1).

**TABLE 1:** Representative empirical studies supporting CCN1 as a compartment-specific biomarker candidate.

Biological context	CCN1 compartment measured	Model/system	Key empirical finding	Biomarker implication
Fracture repair (19).	Tissue/callus expression	Rat fracture callus	CCN1 mRNA and protein were temporally regulated during fracture repair and localized to reparative tissues.	Supports CCN1 as a local marker of active skeletal repair.
Fracture repair and mechanical stability (20).	Callus protein expression	Ovine tibial osteotomy	Early CCN1 upregulation was reduced by decreased fixation stability and associated with reduced vascularization and delayed healing.	Links CCN1 to mechanically regulated repair quality.
VEGF-osteoblast-endothelial coupling (21).	Cell surface, ECM, and soluble osteoblast-associated CCN1	Osteoblast/endothelial assays and mouse fracture model	VEGF induced CCN1 in osteoblasts; CCN1 mediated endothelial migration, sprouting, *in vivo* angiogenesis, and fracture repair.	Supports CCN1 as a mediator/marker of angiogenic repair signaling.
Bone marrow aging (23, 24).	ECM-bound CCN1	Young vs. elderly BM stromal cell-derived ECM and mouse bone	CCN1 was present in young ECM but depleted in elderly ECM; restoring CCN1 improved BM-MSC osteogenic responsiveness.	Supports ECM-bound CCN1 as a skeletal niche biomarker.
Breast cancer liquid biopsy (17).	Soluble plasma CCN1	Plasma ELISA in breast cancer and controls	Circulating CCN1 distinguished breast cancer samples from controls in the reported cohorts and showed pre-analytical stability.	Supports soluble CCN1 as a candidate blood biomarker requiring external validation.
Disseminated/circulating tumor cells (12, 57).	Cell-associated CCN1	DTC lines and patient CTCs	CCN1 was detected in tumor cell populations and CCN1 loss reduced tumor cell viability.	Suggests CCN1 marks survival-associated disseminated tumor cell states.
Pancreatic cancer stroma and immunity (58, 59).	Tumor/stromal CCN1	PDAC experimental models	CCN1 promoted collagen and chemokine programs linked to immune exclusion and therapy resistance.	Supports CCN1 as a remodeling-state marker in immunosuppressive stroma.
Rheumatoid arthritis (46).	Serum CCN1	Clinical RA cohorts	Serum CCN1 was elevated in RA compared with controls but inversely correlated with disease activity.	Demonstrates measurability and context-dependent interpretation.
COPD and inflammation (49).	Serum and pulmonary CCN1	COPD patient samples	CYR61 was associated with pulmonary inflammation and lung function indices.	Highlights limited disease specificity of circulating CCN1.
Cardiovascular injury (60, 61).	Circulating/tissue CCN1	Coronary artery disease/acute coronary syndromes	CCN1 has been investigated as a soluble marker associated with vascular injury.	Suggests circulating CCN1 may reflect systemic injury/remodeling.

## 6 CCN1 orchestrates immune and stromal remodeling to support metastatic persistence in the bone marrow niche

CCN1 is increasingly recognized as a key regulator of immune remodeling within the tumor microenvironment (TME), with important implications for the bone marrow metastatic niche. Through activation of proinflammatory genetic programs, CCN1 promotes macrophage recruitment and polarization, contributing to immune suppression and tumor progression (62–65). In pancreatic ductal adenocarcinoma (PDAC), CCN1 upregulation drives collagen and chemokine expression that facilitate exclusion of cytotoxic T cells while recruiting myeloid-derived suppressor cells (MDSCs), thereby establishing an immunosuppressive microenvironment favorable to tumor growth (66). Conversely, CCN1 deficiency reduces collagen and chemokine expression, enhances cytotoxic immune infiltration, decreases MDSC abundance, and improves therapeutic sensitivity (66). These findings are particularly relevant to bone because the marrow functions as an immune-dense sanctuary for disseminated tumor cells and is highly susceptible to stromal remodeling (57). As an immunologically “cold” tumor, PDAC relies heavily on stromal components to generate a tumor-promoting microenvironment, where collagen-rich extracellular matrix acts as a physical barrier to immune cell infiltration and therapeutic delivery (58, 59). Since CCN1 directly regulates both extracellular matrix deposition and chemokine signaling, its upregulation may similarly condition the bone marrow niche to support immune evasion and metastatic persistence (67–70). Evidence from glioblastoma further supports this concept, as tumor-derived CCN1 enhances macrophage infiltration, promoting tumor growth and suppressing cytotoxic immunity (63–65). Given the central role of macrophages in osteoclast differentiation, bone remodeling, and immune tolerance, analogous CCN1-dependent mechanisms may facilitate survival of circulating and disseminated tumor cells (12, 25, 26, 31, 71, 72). In addition to its role in solid tumors, elevated CCN1 expression within the bone marrow microenvironment promoted leukemic cell survival and therapeutic resistance in acute lymphoblastic leukemia and B-cell acute lymphoblastic leukemia, highlighting a broader role for CCN1 in supporting malignant persistence through immune modulation, stromal conditioning, and resistance to apoptosis-inducing therapies (8, 73, 74).

## 7 Clinical and therapeutic implications

The clinical value of CCN1 depends not on whether it functions as a biomarker in the broadest sense, but rather on which CCN1 pool is being measured, in what disease context, and against which clinical comparator. A practical translational framework includes: (1) tissue- or extracellular matrix (ECM)-associated CCN1 as a local indicator of matrix remodeling, osteogenesis, and tissue repair; (2) soluble CCN1 as a blood-accessible marker of systemic injury, inflammation, or tumor-associated remodeling; and (3) CCN1-positive circulating tumor cells as a marker of disseminated tumor cell states. Such compartment-specific interpretation may help reconcile seemingly contradictory findings across cancer and non-cancer diseases while improving biomarker study design and clinical translation. In cancer-associated bone pathology, a particularly compelling hypothesis is that CCN1 could serve as a marker of active metastatic niche remodeling, identifying patients with ongoing tumor–stroma interactions when used alongside established tumor and bone biomarkers. Although CCN1 is implicated in cardiovascular injury, chronic obstructive pulmonary disease, rheumatoid arthritis, and other inflammatory conditions, its specificity may be enhanced through careful cohort selection, integration with complementary biomarkers, and multivariable predictive modeling. Importantly, successful clinical implementation will require standardization of pre-analytical variables, including specimen collection, processing, storage conditions, freeze–thaw cycles, and assay platforms. Longitudinal studies should evaluate whether changes in circulating CCN1 precede imaging-detectable skeletal progression, correlate with bone turnover markers, or predict response to therapies targeting tumor–stroma interactions, immune pathways, angiogenesis, or bone remodeling.

Clinical feasibility is supported by studies demonstrating the stability of CCN1 in plasma and its discriminatory performance in breast and lung cancer detection, while liquid biopsy approaches have established that tumor-derived proteins and other circulating tumor components can be reliably measured in blood (12, 13, 17, 60, 61, 75–77). ELISA-based studies further show significantly elevated CCN1 levels in breast cancer samples compared with benign breast disease and acute cardiac controls, supporting its potential utility for cancer detection and disease monitoring (17, 60, 61).

Beyond its biomarker potential, CCN1 represents an attractive therapeutic target because of its central role in extracellular matrix remodeling, inflammation, angiogenesis, immune regulation, and treatment resistance. Current strategies under investigation include CCN1 gene silencing, disruption of CCN1–integrin signaling, and combination approaches designed to overcome chemoresistance (37–39, 78). Emerging therapeutic technologies, including chimeric antigen receptor T-cell (CAR-T) therapies and nanotechnology-based delivery systems, may create new opportunities to target CCN1-dependent signaling networks and the tumor microenvironment in cancer (79, 80). However, therapeutic development must account for CCN1’s dual biology as both a matrix-bound and soluble signaling molecule, as well as its context-dependent functions in tissue repair and homeostasis. Selective targeting of pathogenic CCN1 activity while preserving its physiological roles will likely require compartment-specific and cell-type–specific approaches. Collectively, current evidence positions CCN1 as a promising biomarker and therapeutic target whose dynamic regulation across tumor, stromal, immune, and bone microenvironments may provide new opportunities for cancer screening, risk stratification, treatment monitoring, and precision therapeutic intervention (12, 13, 17, 37–39, 44, 60, 61, 75–78, 81).

## 8 Conclusions and future perspectives

CCN1 has emerged as a central regulator at the convergence of angiogenesis, bone remodeling, and immune modulation, positioning it as a key mediator of tissue homeostasis and pathological remodeling. Its multifunctional, integrin-dependent signaling network illustrates how physiological programs involved in development, repair, and regeneration can be co-opted during tumor progression and metastatic bone disease. Furthermore, the existence of both ECM-bound and soluble CCN1 broadens its biological influence and highlights its potential translational value as both a therapeutic target and a circulating biomarker.

**FIGURE 1:**
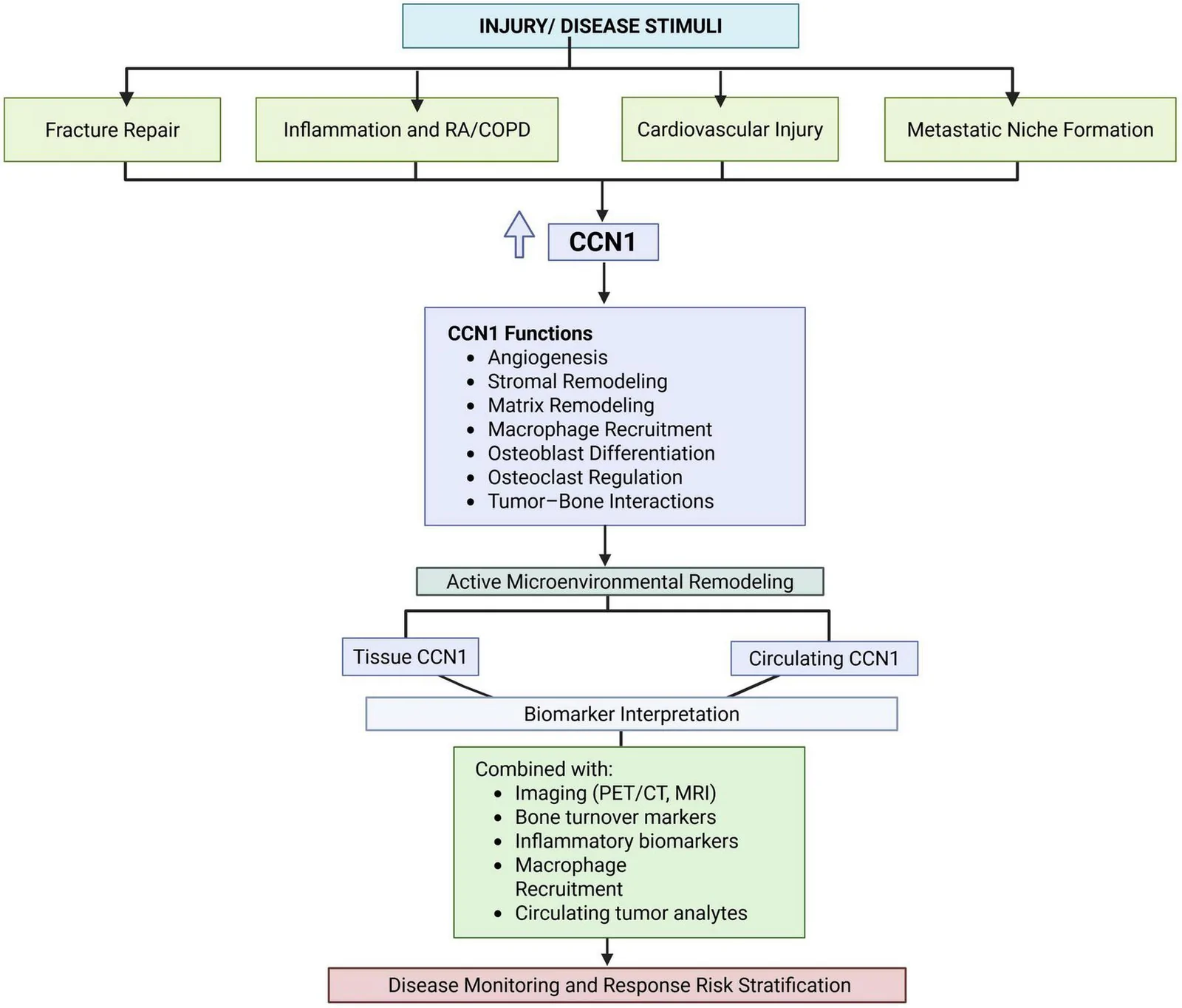
Cellular communication network factor 1 (CCN1) as a dynamic biomarker of microenvironmental remodeling in bone pathology and skeletal metastasis. A conceptual model illustrating CCN1 as a central mediator and biomarker of tissue remodeling. Physiological and pathological stimuli, including fracture repair, inflammation, cardiovascular injury, and metastatic niche formation, induce CCN1 expression in stromal, endothelial, inflammatory, and tumor-associated cells. Through interactions with integrins and extracellular matrix components, CCN1 regulates angiogenesis, immune cell recruitment, matrix remodeling, osteogenesis, and osteoclast activity, thereby shaping local tissue microenvironments. These remodeling programs generate measurable circulating and tissue-associated pools of CCN1 that may reflect ongoing biological activity rather than a specific disease process. The distribution of CCN1 between tissue-associated and circulating pools is likely dynamic and influenced by secretion, ECM-binding capacity, integrin/HSPG availability, proteolytic remodeling, mechanical cues, inflammation, and tumor-associated release. Consequently, the greatest translational value of CCN1 may lie in its integration with imaging modalities, bone turnover markers, inflammatory biomarkers, and tumor-derived analytes to improve interpretation of disease activity and therapeutic response. This framework supports the use of CCN1 as a dynamic indicator of active microenvironmental remodeling in bone disease and skeletal metastasis rather than as an isolated diagnostic marker. Created in BioRender. Ortiz, G. (2026) https://BioRender.com/bzbioqe.

Collectively, the available evidence supports a nuanced and biologically informed framework for interpreting CCN1 as a compartment-specific indicator of active tissue remodeling and tumor–microenvironment interactions. Rather than viewing CCN1 as a universal biomarker, current data underscore the importance of distinguishing among ECM-tethered, soluble, and tumor cell-associated pools of CCN1. This conceptual distinction helps explain the protein’s relevance across diverse processes, including fracture healing, skeletal aging, inflammatory and vascular disease, and cancer dissemination, while emphasizing the need for context-dependent interpretation of biomarker measurements (Figure 1).

Future investigations should focus on defining the receptor-specific and compartment-dependent mechanisms through which CCN1 regulates tumor–bone crosstalk, with the goal of developing interventions that selectively attenuate metastatic progression while preserving physiological tissue repair and regenerative functions (14, 15, 19, 22, 24, 32, 42–45, 60, 61, 66, 82). Equally important will be the longitudinal validation of CCN1 in skeletal disorders and cancer-associated bone pathology using standardized analytical platforms and integrated assessment of tissue, circulating, imaging, and tumor-derived endpoints. Such studies will be critical for determining the clinical utility of CCN1 as a biomarker of disease activity and a potential target for therapeutic intervention in metastatic bone disease.
